# Supplementary figures and images for: Fish mislabelling in France: substitution rates and retail types
Source: PeerJ. 2015 Jan 6;2:e714. doi: 10.7717/peerj.714 (PMC5407283; doi:10.7717/peerj.714)

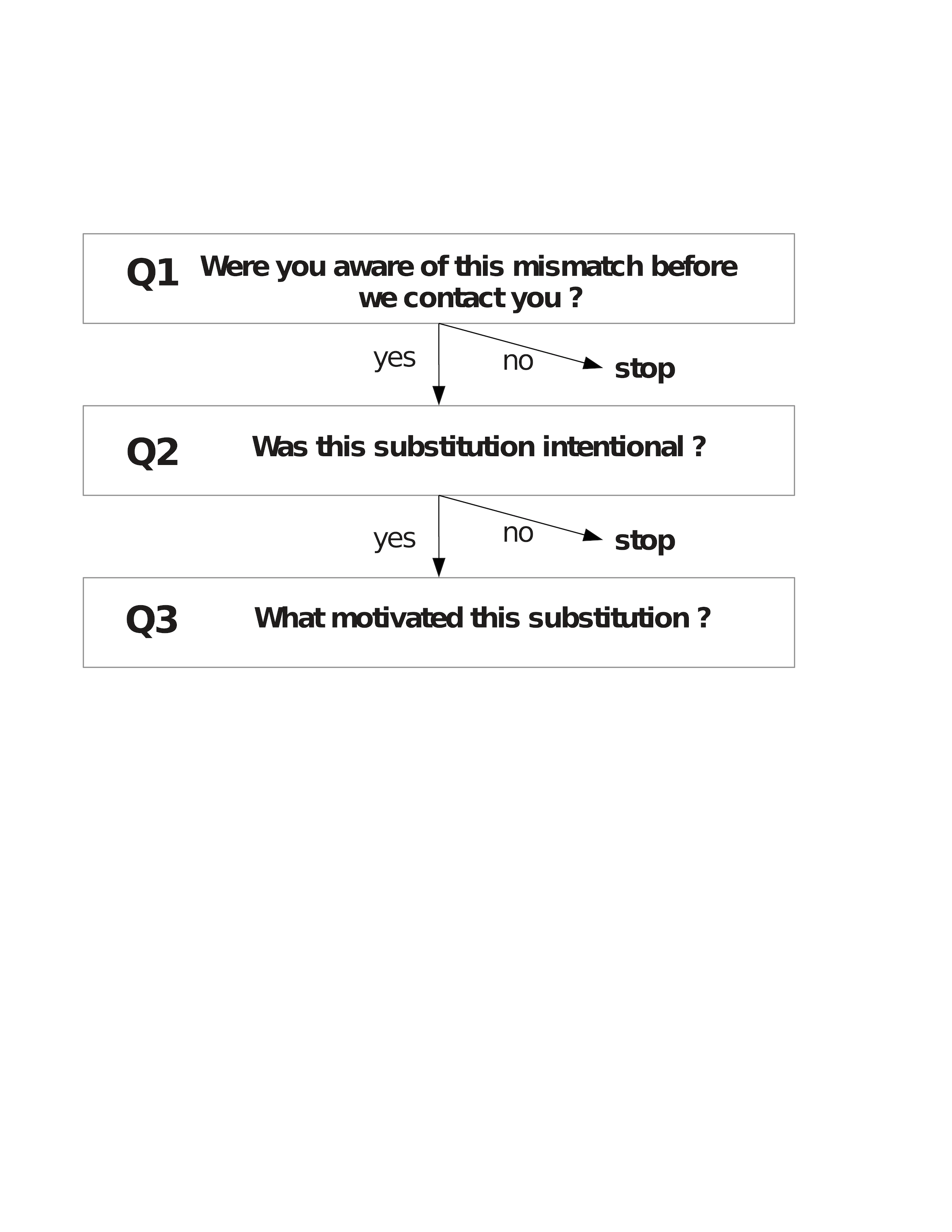

Supplement: Figure S1 — The questions were asked in order, either over the phone or face to face. [file peerj-03-714-s001.png]

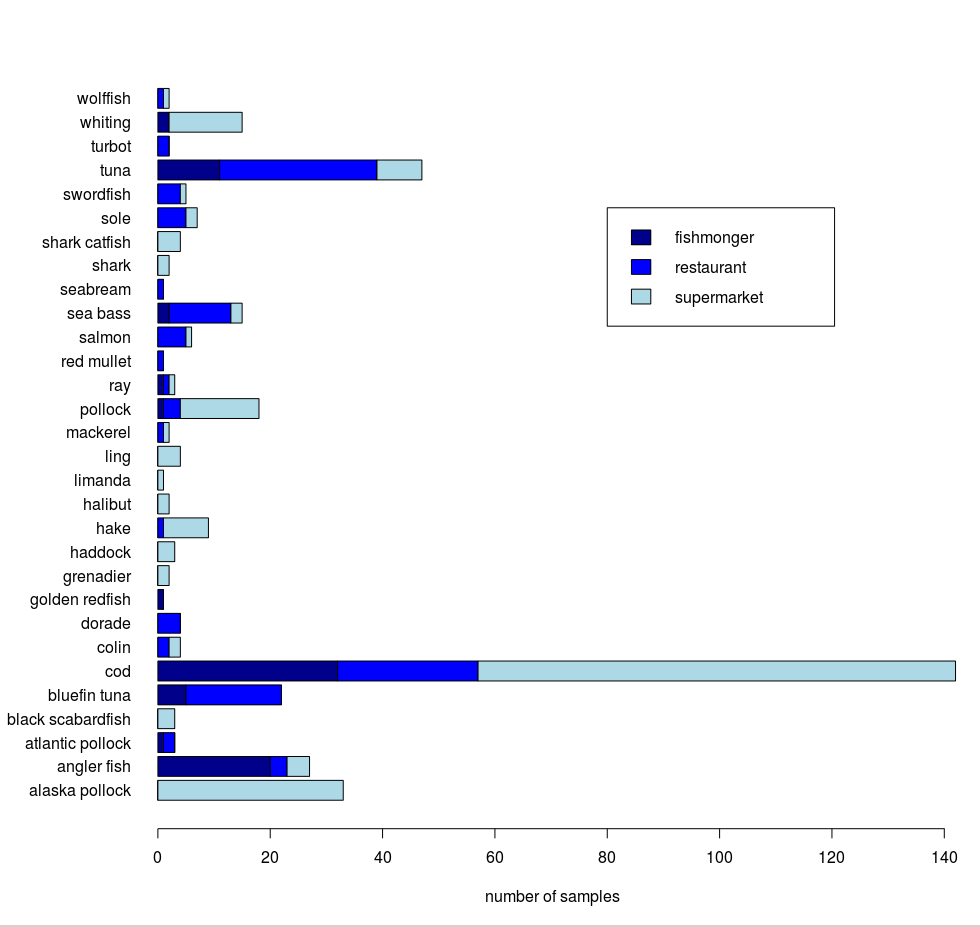

Supplement: Figure S2 — Commercial names indicated on the y-axis represent the same species or closely related species. A few account for most of the samples (cod, tuna, alaska pollock and anglerfish) while a long tail of species include less than 4 samples. For the statistical analysis, only categories with more than 10 samples were kept and the rest were grouped together. [file peerj-03-714-s002.png]

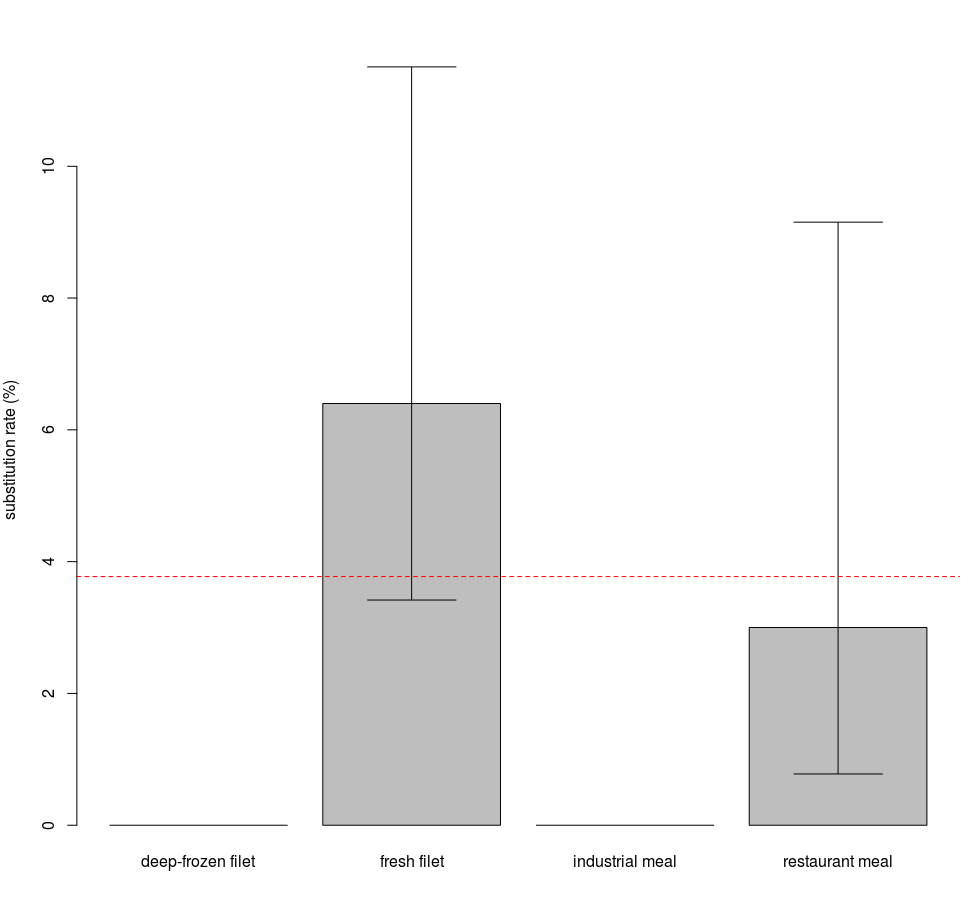

Supplement: Figure S3 — Mislabelling was detected only for fresh fillets and restaurants meals. Error bars show the 95% confidence interval. The red dashed line is the average substitution rate observed for the entire dataset. [file peerj-03-714-s003.png]
